# Impaired IFN-α-mediated signal in dendritic cells differentiates active from latent tuberculosis

**DOI:** 10.1371/journal.pone.0189477

**Published:** 2018-01-10

**Authors:** Stefania Parlato, Teresa Chiacchio, Debora Salerno, Linda Petrone, Luciano Castiello, Giulia Romagnoli, Irene Canini, Delia Goletti, Lucia Gabriele

**Affiliations:** 1 Department of Oncology and Molecular Medicine, Istituto Superiore di Sanità, Rome, Italy; 2 Translational Research Unit, Department of Epidemiology and Preclinical Research, "L. Spallanzani" National Institute for Infectious Diseases (INMI) IRCCS, Rome, Italy; 3 Center for Life Nano Science@Sapienza, Istituto Italiano di Tecnologia, Rome, Italy; 4 Istituto Pasteur-Fondazione Cenci Bolognetti, Rome, Italy; Rutgers Biomedical and Health Sciences, UNITED STATES

## Abstract

Individuals exposed to *Mycobacterium tuberculosis* (*Mtb*) may be infected and remain for the entire life in this condition defined as latent tuberculosis infection (LTBI) or develop active tuberculosis (TB). Among the multiple factors governing the outcome of the infection, dendritic cells (DCs) play a major role in dictating antibacterial immunity. However, current knowledge on the role of the diverse components of human DCs in shaping specific T-cell response during *Mtb* infection is limited. In this study, we performed a comparative evaluation of peripheral blood circulating DC subsets as well as of monocyte-derived Interferon-α DCs (IFN-DCs) from patients with active TB, subjects with LTBI and healthy donors (HD). The proportion of circulating myeloid BDCA3^+^ DCs (mDC2) and plasmacytoid CD123^+^ DCs (pDCs) declined significantly in active TB patients compared to HD, whereas the same subsets displayed a remarkable activation in LTBI subjects. Simultaneously, the differentiation of IFN-DCs from active TB patients resulted profoundly impaired compared to those from LTBI and HD individuals. Importantly, the altered developmental trait of IFN-DCs from active TB patients was associated with down-modulation of IFN-linked genes, marked changes in molecular signaling conveying antigen (Ag) presentation and full inability to induce Ag-specific T cell response. Thus, these data reveal an important role of IFN-α in determining the induction of *Mtb-*specific immunity.

## Introduction

Individuals infected with *Mycobacterium tuberculosis* (*Mtb*) may remain latently infected for years or develop active tuberculosis (TB). Among the multiple factors governing infection outcome, recent evidences support a non-redundant role of CD8^+^ T cells [1, 2]. Dendritic cells (DCs) are major players in cross-processing exogenous antigens (Ags), including those of *Mtb*, to CD8^+^ T-cells [3]. DC family comprises phenotypically and functionally specialized subsets such as plasmacytoid DCs (pDCs) and conventional DCs (cDCs) [4]. In human blood and peripheral lymphoid organs, cDCs consist of myeloid BDCA1^+^ DCs (mDC1) and BDCA3^+^ mDC2, driving CD4^+^ T cell proliferation and cross-presenting Ags from pathogens and dead cells, respectively [4]. Likewise, blood CD123^+^ pDCs efficiently cross-present soluble viral and cell-associated antigens (Ags) [5]. However, upon activation with specific signals, mDC1s and mDC2s may display comparable efficiency in cross-presentation [4]. To date, few studies have assessed the role of DC subsets in the immune response against *Mtb*. Untreated acid-fast bacilli (AFB)-smear positive pulmonary tuberculosis (TB) patients have higher pDCs and lower mDCs compared with healthy family contacts [6]. Moreover, the generally rare pDCs highly infiltrate the skin in individuals positive for tuberculin skin test [7]. Of interest, a crosstalk between pDCs and BDCA1^+^ cDCs has been recently reported to promote antibacterial activity and CD4^+^ T-cell stimulation against Bacille Calmette-Guerin (BCG) [8].

Precursors of DCs can also be monocytes [9]. Interferon (IFN)-α induces *in vitro* one-step differentiation of monocytes into highly activated and partially mature IFN-DCs, characterized by marked phagocytic activity and special attitude in inducing CD8^+^ T-cells against both viral and tumor Ags [10, 11]. Many studies on IFN-DCs have led to the concept that these cells resemble naturally occurring DCs, rapidly generated from monocytes in response to danger signals, including infections [12]. Recently, we have shown that IFN-DCs and pDCs share common phenotypic, molecular and functional properties [13]. Beyond the expression of the same microRNA pattern and comparable levels of pDC-associated markers such as CD123, CD2AP, TLR7, TLR9 and IRF-8, both populations display strong ability to cross-prime CD8^+^ T cells upon MHC-I rearrangement and to produce type I IFN (IFN-I) upon infection. To date, the role of the diverse DC components in *Mtb* Ag-presentation remains to be clarified.

IFN-I stimulate DCs to cross-present Ags and activate CD8^+^ T cells [14]. Although IFN-I appear to promote rather than control TB, their role in the immune response to *Mtb* infection remain unclear [15, 16]. While IFN-I-induced genes dominate whole blood transcriptional profile of TB patients with a strong disease-severity correlation [17], IFN-I may also have beneficial effects mediating innate immune control of early BCG infection, via DC maturation [18, 19]. *Mtb* inhibits IFN-I induction and Ag cross-processing through TLR9 signaling [20], confirming host advantage for IFN-I activity in some TB settings. Of interest, TB patients exhibit impaired production of IFN-α by peripheral blood mononuclear cells (PBMC) upon stimulation and decreased circulating mDC and pDC levels [6, 21].

In the present study, we performed a comparative characterization of circulating DCs in active TB and individuals with latent TB infection (LTBI) compared to healthy donors (HD). Moreover, we characterized IFN-DCs from active TB, LTBI and HD individuals at phenotypic, transcriptional and functional levels. We found that impaired IFN-α signal in DCs from active TB patients might account for their inability to generate T cell response against *Mtb*, which is conversely retained in LTBI subjects.

## Materials and methods

### Patient population

National Institute of Infectious diseases (INMI) “L.Spallanzani” Ethical Committee approved this study (number 02/2007). A draw blood of 17 ml was performed in the enrolled individuals that provided written informed consent (n = 54; Table 1). Active TB patients, diagnosed with drug-susceptible pulmonary TB by *Mtb* sputa positive culture, were enrolled within 7 days starting specific treatment. LTBI donors, reporting remote or recent contact with smear-positive active TB patients [22], were selected for positive score to Quantiferon-Gold in Tube (QFT-IT) (Qiagen) without lung TB lesions [23]. All enrolled subjects were HIV-uninfected, not under immunosuppressive treatments, nor with diabetes [24, 25]. HD subjects, with QFT-IT-negative score, reported no exposure to *Mtb*. Demographic and epidemiological information were collected.

**Table 1:** Demographic characteristics of enrolled subjects.

	Active TB	LTBI	HD	Total	*P* value
N (%)	21 (39)	18 (33)	15 (28)	54 (100.0)	
**Median Age (IQR)**	37 (28.0–45.0)	43 (32.0–50.0)	39 (30.0–48.0)	40 (30.0–48.0)	0.438 ^a^
**Male gender (%)**	13 (62)	12 (67)	4 (27)	29 (54)	0.045 ^b^
**Origin (%)**					<0.0001 ^b^
**Western Europe**	2 (10)	8 (44)	15 (100.0)	25 (46)	
**Eastern Europe**	12 (57.1)	7 (38.9)	0 (0)	19 (35.1)	
**Africa**	4 (19.0)	1 (5.6)	0 (0)	5 (9.3)	
**South America**	3 (14.3)	2 (11.1)	0 (0)	5 (9.3)	
**QFT-IT (%)**					NA
**Positive**	18 (86)	18 (100.0)	0 (0)	36 (67)	
**Negative**	3 (14.3)	0 (0)	15 (100.0)	18 (33.3)	
**Indeterminate**	0 (0)	0 (0)	0 (0)	0 (0)	
**BCG status (%)**					<0.0001 ^b^
**Vaccinated**	19 (91)	10 (56)	3 (20.0)	32 (59)	
**Unvaccinated**	2 (9)	8 (44)	12 (80.0)	22 (41)	

HD: healthy donor; Active TB: tuberculosis; LTBI: latent tuberculosis infection; N: number; IQR: interquartile range; QFT-IT: QuantiFERON TB-Gold in tube; BCG: Bacillus Calmette et Guérin; NA: not available.

^**a**^ Kruskal-Wallis test.

^**b**^ Chi-Square test

### Identification of circulating DC subsets

For DC subsets analysis, one-hundred microliters of whole peripheral blood, collected in 6 ml heparinized Vacutainer tubes (BD Bioscience), were directly stained with antibodies (Abs): anti-Lineage(Lin)-cocktail-(FITC), anti-HLA-DR-(Per-CPCy5.5), anti-CD11c-(HorizonV450), anti-CD86-(APC), anti-CD123-(PE), anti-BDCA-3-(PE), anti-CD80-(APC-H7) (all from BD Bioscience). After staining, red cells were lysed with FACS Lysing Solution (BD Biosciences). Samples were acquired by FACS CantoII cytometer (BD Bioscience) and data were analyzed using FlowJo software (Tree Star Inc.) Fluorescence-minus-one controls were performed to assess the spread of all the fluorophores into the missing channel, and to set the gates accordingly.

### Cell culture and stimuli

IFN-DCs were generated as previously described [26]. Briefly, CD14^+^ monocytes were isolated from peripheral blood by positive magnetic immunoselection (CD14^+^ Microbeads, Miltenyi Biotec) and cultured for 3 days with 500 U/ml GM-CSF (PeproTech) and 10^4^ IU/ml IFNα2b (Intron A, Merck Sharp and Dohme). IFN-DCs were analyzed for CD80, CD86, BDCA1, BDCA3 and CD123 expression by FACSCalibur (all from BD Biosciences). In some experiments, IFN-DCs were stimulated for 24 hr with recombinant proteins ESAT-6/CFP-10 (hereafter RD1) (25 μg/ml each) (Lionex), purified protein derivative (PPD) (50 μg/ml) [22] or Staphylococcal enterotoxin B (SEB) (2μg/ml) as positive control. Autologous total lymphocytes were isolated by negative magnetic immunoselection, simultaneously to the isolation of CD14^+^ monocytes.

### T cell response to TB Ags and cytokine detection

RD1, PPD or SEB-loaded IFN-DCs were co-cultured in triplicate with autologous T cells, at different stimulator/responder (E/T) ratios. After 6-day co-culture, IFN-DC capability to induce Ag-specific T cell proliferation was evaluated by methyl-3H incorporation. IFN-γ, TNF-α, IL-6 and IL-2 secretion was evaluated in supernatants of RD1-loaded IFN-DCs/autologous T cells co-cultures after 24 and 72 hr by multiplexed bead-based immunoassay (Cytometric Bead Array, BD Bioscience).

### Microarray hybridization and data mining

Microarray chips were made in-house by spotting on poly-L-lysine-coated glass slides (SuperChip I, Perkin Elmer) 34,580 70mer oligonucleotides (Human Genome Oligo Set version 3.0, Operon) by means of OmniGrid microarrayer (Gene Machines/Genomic Solution). Total RNA from IFN-DCs was extracted (RNAeasy kit, QIAGEN) and quality assessed by using NanoDrop ND-1000 UV-Vis Spectrophotometer (Thermo Scientific, USA) and Agilent 2100 bioanalyzer (Agilent Technologies). Cy5-labeled aRNA, obtained by T7 RNA polymerase-mediated amplification, was co-hybridized with reference aRNA (Universal Human Reference RNA, Stratagene) onto chips. Slides were scanned by Gene Pix 4200 (Molecular Devices); microarray images and data files were analyzed by ScanArray Software. The dataset is represented by 5 test arrays for each condition (TB = 5; LTBI = 5; HD = 5). Class comparison (Two sample t-test with a randomized variance model) was performed by BRB-ArrayTools software (BRB-ArrayTools Development Team). Differentially expressed genes were selected with *P*≤0.005 threshold. Heat maps were generated by CLUSTER and Java TREEVIEW software [27, 28]; Principal Component Analysis (PCA) and GeneSet enrichment analysis (GSEA) were performed using R and GSEA software and Broad Institute Molecular Signatures Database, respectively [29, 30]; IFN signature was analyzed by BROWNE_INTERFERON_RESPONSIVE_GENES GeneSet (http://software.broadinstitute.org/gsea/msigdb/cards/); biological functions, gene networks and canonical pathways were identified by Ingenuity pathway core analysis (IPA) (IPA^®^, QIAGEN Redwood City, www.qiagen.com/ingenuity).

Microarray data were deposited into Gene Expression Omnibus database (GEO) (www.ncbi.nlm.nih.gov/geo) (accession#GSE84344). Further information is available from the authors upon request.

### Quantitative RT-PCR

RNA was extracted by RNeasy mini kit (Qiagen) and analyzed for quality by means of NanoDrop ND-1000. cDNA-reverse transcribed (cDNA synthesis kit, Bioline) was assayed by quantitative RT-PCR (SensiMix Plus SYBR kit, Quantace) using the oligonucleotides: CD1a (5’-CGCACCATTCGGTCATTTGAGG, 3’-TCCTGAGACCTTTCCAGAGTGC), CD80 (5’CTCTTGGTGCTGGCTGGTCTTT, 3’-GCCAGTAGATGCGAGTTTGTGC), LAMP3 (5’-TGAAAACAACCGATGTCCAA, 3’-TCAGACGAGCACTCATCCAC), LGALS2 (5’-GGGCAAGAACAACGGGAAGATC, 3’-CCTGTTGGGAAAAGTCAGCTCG). Amplification was performed by 7500 qPCR System (Applied biosystems); mRNA relative expression was normalized to β-actin by comparative 2^-ΔCt^ method.

### Statistical analysis

Statistical analyses of experiments on peripheral DCs were performed using STATA (Version 8.0) or SPSS (Windows-Version 19) software. Wilcoxon test was used for two-tailed paired samples. Pairwise and multiple groups comparisons were performed by Mann-Whitney U test and Kruskal Wallis, respectively. Bonferroni correction was used if needed. *P* values <0.05 or 0.016 (3 groups) were considered significant. Statistical analysis of proliferation experiments and molecular data were performed using Mann-Whitney U test. Graphpad Prism v.6.01 and Microsoft Excel were used to edit data. *P* values <0.05 were considered statistically significant. *P<0.05; **P<0.01; ***P<0.001.

## Results

### Decline in BDCA3^+^mDC2 and CD123^+^pDCs in active TB patients contrasts with DC activation in LTBI subjects

**Fig 1:**
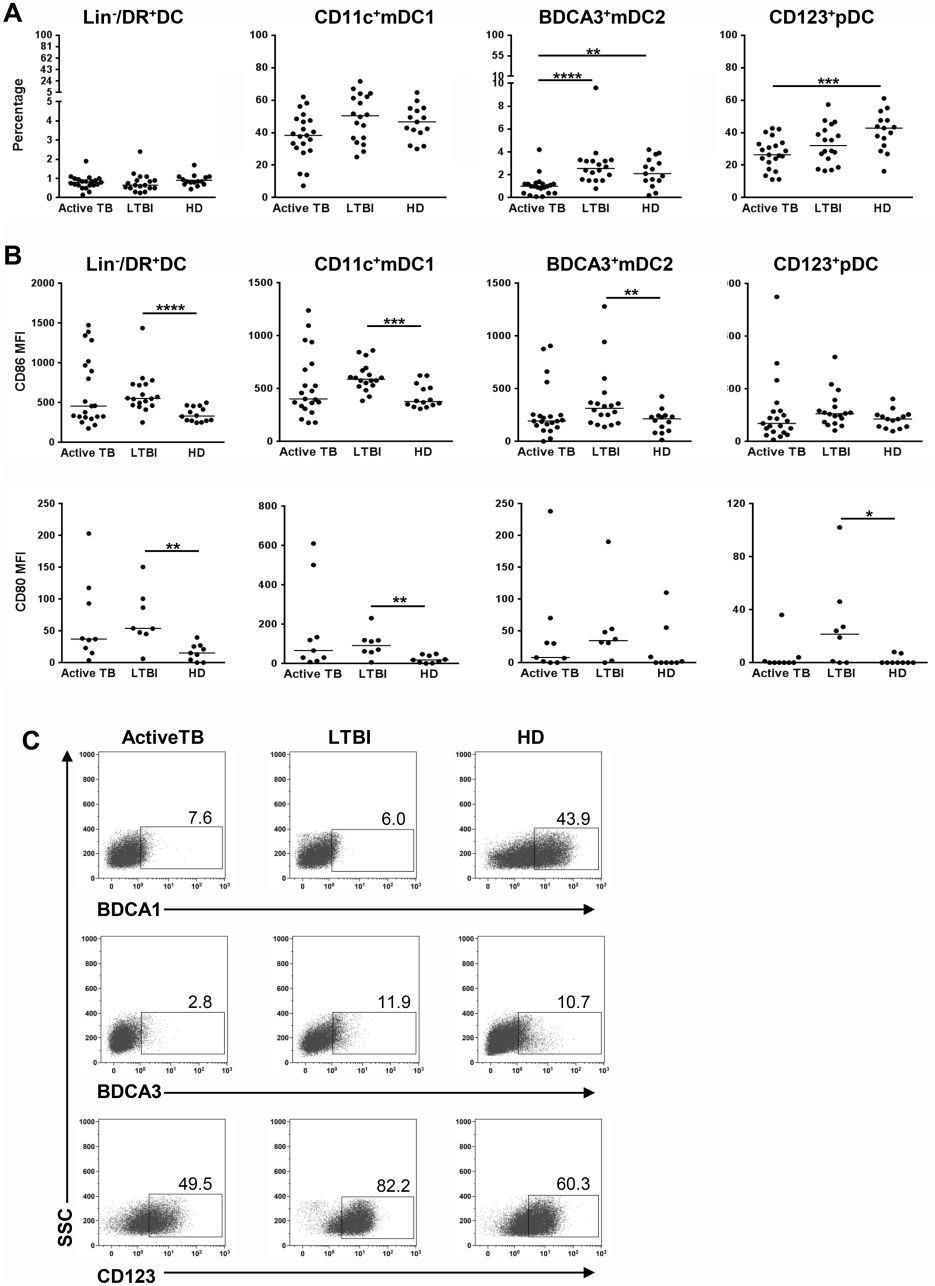
Analysis of circulating DC subsets in peripheral blood of active TB, LTBI or HD individuals. *A*, Percentage of peripheral blood Lin^-^/HLA-DR^+^ DCs, CD11c^+^ mDC1, BDCA3^+^ mDC2 and CD123^+^ pDCs were determined by flow cytometry. Median values are shown. *B*, CD86 and CD80 expression on total DCs and DC subsets are shown. Data are reported as mean fluorescence intensity (MFI) and median values are indicated. Statistical significance was analyzed by Mann-Whitney test using Bonferroni correction. *P<0.05; **P<0.01; ***P<0.001; ****P<0.0001. *C*, Expression of the type-1 and type-2 myeloid DC markers, BDCA1 and BDCA3, and the plasmacytoid marker CD123 in IFN-DCs derived from active TB, LTBI and HD individuals by flow cytometry. Active TB, LTBI and HD indicate IFN-DCs derived from active TB, LTBI and HD individuals, respectively. Percentages of positive cells are shown. SSC, side scatter. Data are representative of three independent experiments (N = 3).

The frequency of peripheral blood DC subsets was evaluated by multicolor flow-cytometry within circulating Lin^-^/HLA-DR^+^ DCs into cDCs (CD11c^+^/BDCA3^-^mDC1 and CD11c^+^/BDCA3^+^ mDC2) and CD11c^-^/CD123^+^ pDCs (hereafter: CD11c^+^ mDC1, BDCA3^+^ mDC2 and CD123^+^ pDCs, respectively) (S1 Fig). Although total DC percentage was similar in active TB, LTBI and HD subjects, significant differences were observed in DC subsets. Indeed, while LTBI individuals possessed the amount of all three circulating DC subsets as much as HD, active TB patients showed significant lower percentages of BDCA3^+^ mDC2 and CD123^+^ pDCs compared to HD. Notably, the amount of active TB BDCA3^+^ mDC2 in active TB patients was significantly lower also compared to LTBI subjects (Fig 1A). Moreover, LTBI circulating DCs exhibited broadly significant higher levels of CD86 and CD80 co-stimulatory molecules compared to HD counterpart, whereas no difference was observed among active TB and HD circulating DC subsets, suggesting selective activation of LTBI circulating DCs (Fig 1B).

### Impaired differentiation and molecular profiling distinguish IFN-DCs derived from active TB *versus* LTBI individuals

**Fig 2:**
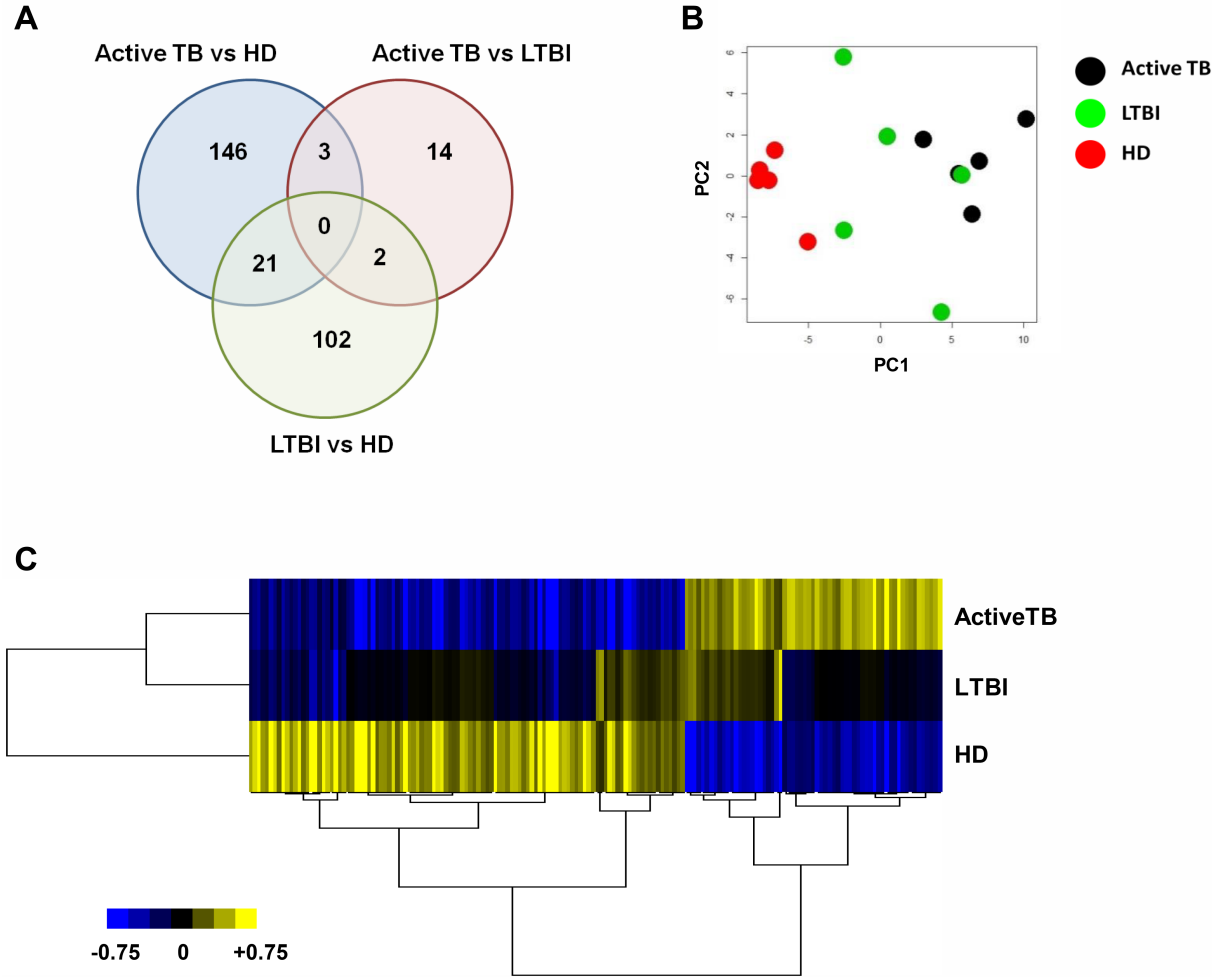
Analysis of differentially expressed genes across active TB, LTBI and HD individuals. Hereafter, active TB, LTBI and HD indicate TB-DCs, LTBI-DCs and HD-DCs, respectively. *A*, Venn’s diagram showing the number of transcripts modulated and their overlaps across IFN-DCs derived from active TB, LTBI and HD individuals. Genes selected on the threshold of *P* <0.005 by Student’s t-test from five biological replicates (n = 5) for each group were included in the analysis. *B*, PCA of differentially expressed genes across active TB, LTBI and HD; PC1 and PC2 were chosen as the axes explicating most of the data variance. *C*, Heat map showing differential expression across active TB, LTBI and HD, built on the selected 170 genes differentially expressed between active TB and HD individuals. The expression level average of five samples for each group is shown. Values are average corrected and shown on a blue-black-yellow gradient: yellow, increased expression; blue, decreased expression.

**Fig 3:**
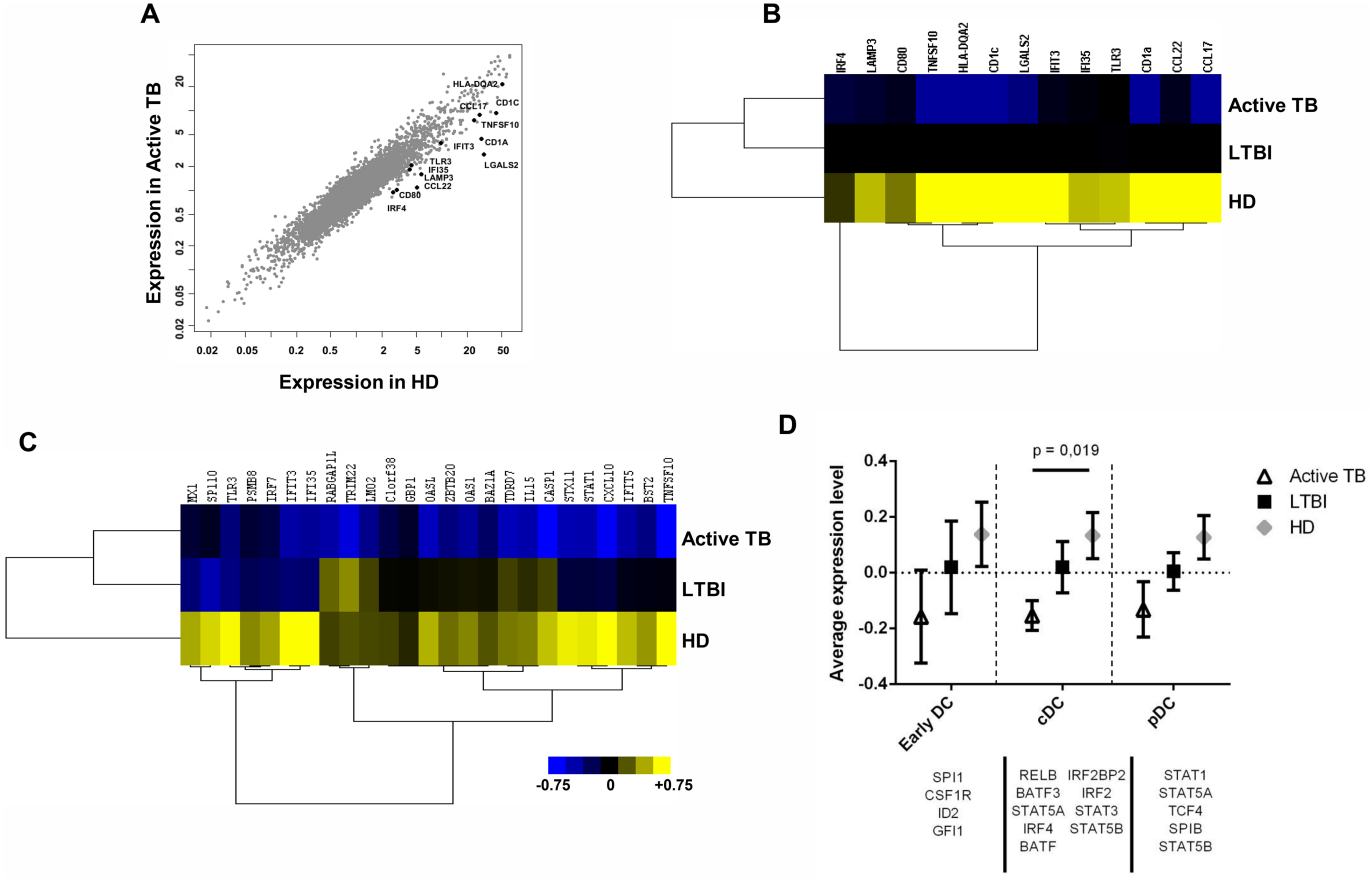
Downregulation of IFN-target genes in IFN-DCs derived from active TB patients. *A*, Scatter plot of gene expression levels between active TB and HD groups showing downregulation of selected genes in active TB compared to HD. *B*, Heat map of selected genes downregulated in active TB compared to HD; median centered Log2 values are shown. Expression values of LTBI are also included. *C*, Hierarchical clustering of 25 genes belonging to IFN signal differentially expressed among active TB, LTBI and HD, built on mean corrected values. In both *B* and *C*, values are expressed on a blue-black-yellow gradient. Yellow, increased expression; blue, decreased expression. *D*, Mean centered expression levels of genes coding transcriptional factors regulating DC development. Statistical significance was analyzed by Student’s t-test (n = 5/group).

**Fig 4:**
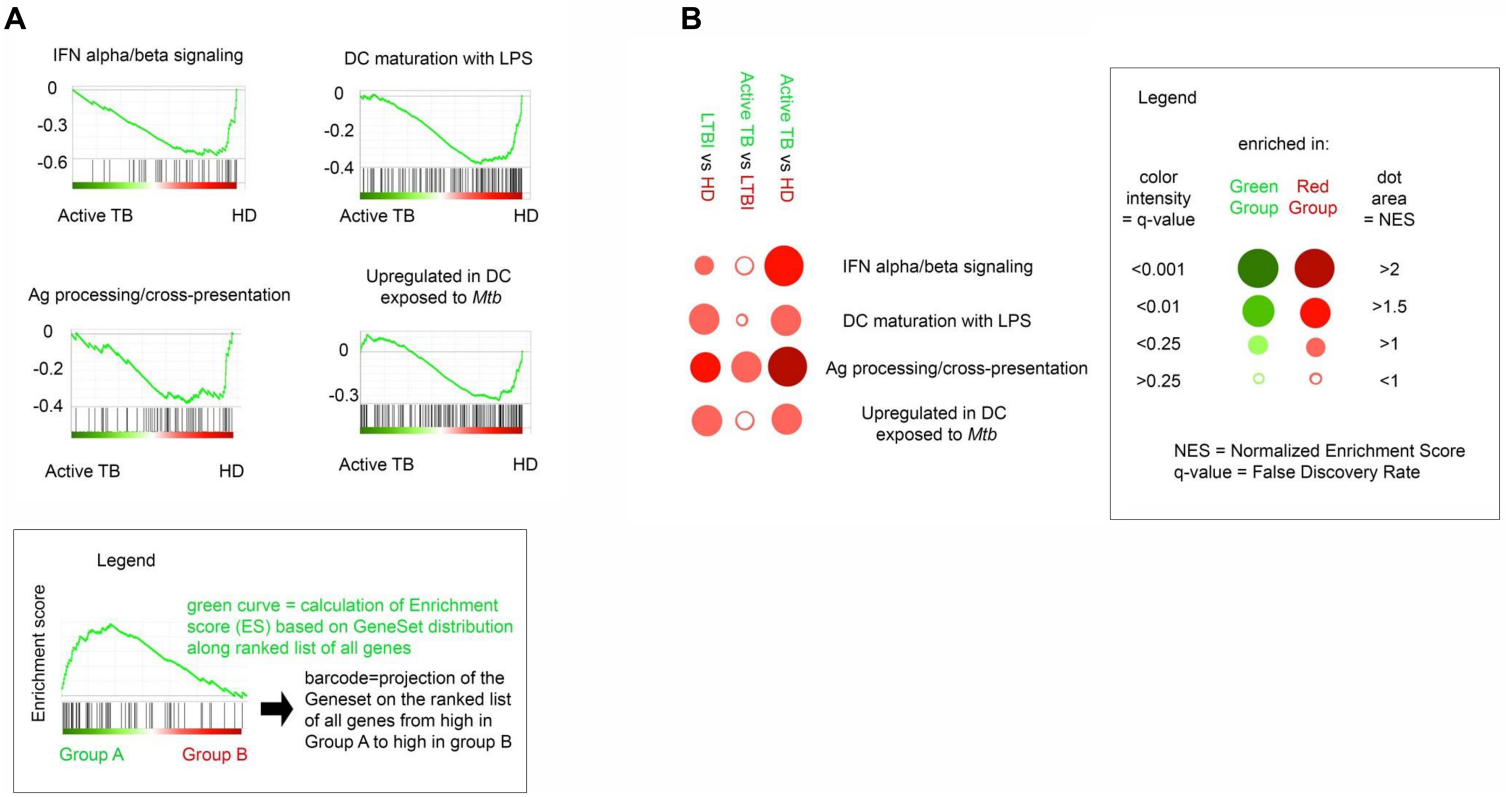
IFN-DCs derived from active TB patients are impaired for DC-related response gene signatures. *A*, GSEA results for comparison between active TB and HD. GeneSets relevant to DC biology are shown. Plot of the running sum for enrichment score (ES) in selected data set, the normalized enrichment score (NES) and location of genes (hits) in the list ranked according to expression differences (barcode) are shown as explained in the legend. *B*, Dot plot representation of GeneSet expression level in comparisons among active TB, LTBI and HD groups, as expressed by NES and q-value. As detailed in the legend, dot area is proportional to NES (<1 = no enrichment; >2 = maximum expression); dot color intensity is proportional to q-value significance, dot color indicates the group in which the GeneSet is overexpressed.

**Fig 5:**
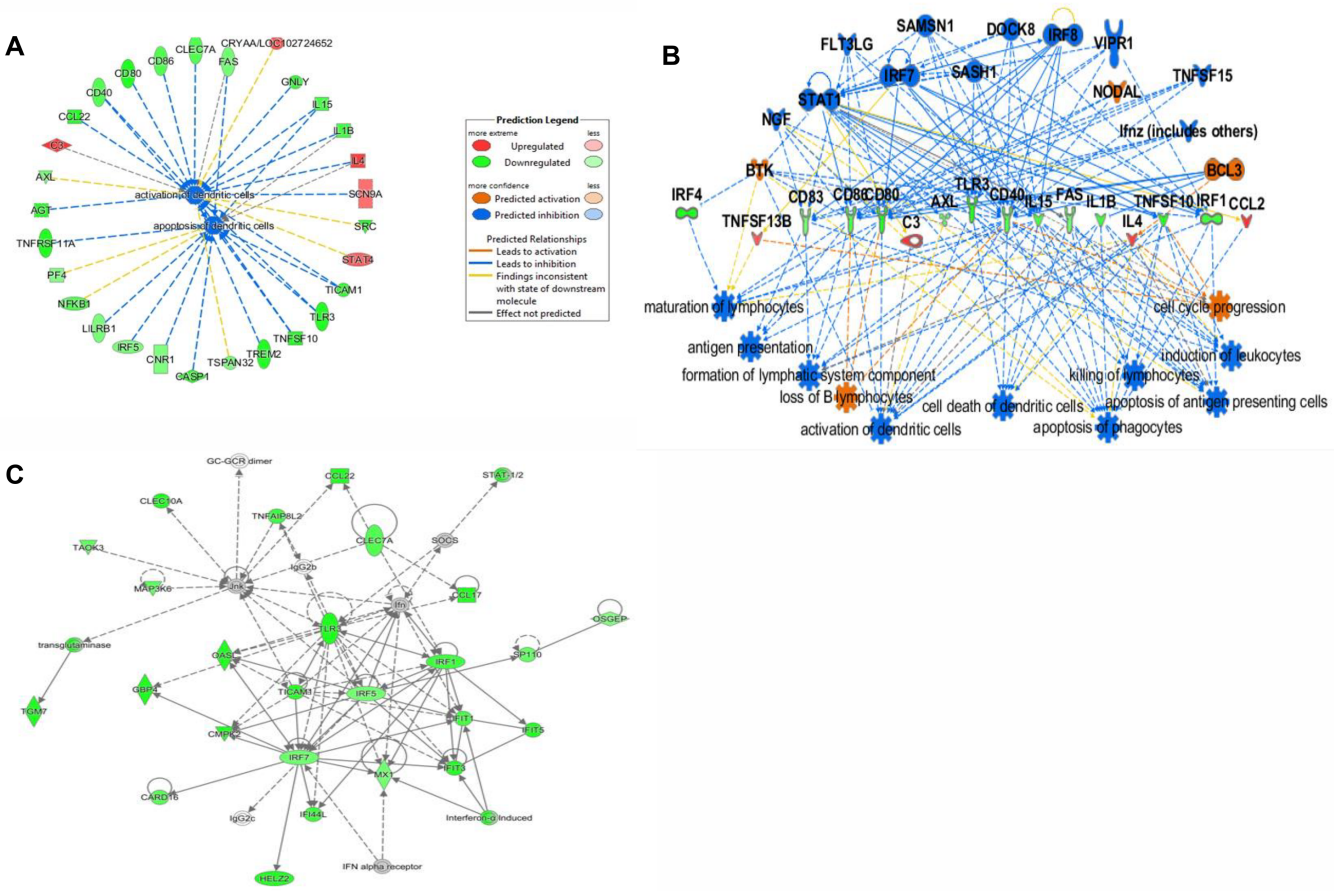
IPA analysis identifies specific impaired functional terms in IFN-DCs derived from active TB patients. Gene lists of 1.5-fold differentially expressed genes in TB-DCs *versus* HD-DCs were mapped by IPA software, revealing the modulation of several pathways and genes. *A*, Genes forming “activation dendritic cells” and “apoptosis dendritic cells” annotation found to be affected in TB-DCs. The direction of change is color coded with green and red indicating down-regulation and up-regulation, respectively. *B*, Regulator Effect Analysis of TB-DCs indicating upstream regulators (at the top) and downstream effects (at the bottom) hypothesized by IPA based on expression level of key genes (in the middle). Color legend as in *A*. *C*, Network of genes most significantly altered in TB-DCs. Genes in green showed a 1.5 fold down-regulation compared to HD-DCs.

The differentiation program of IFN-DCs generated from active TB, LTBI and HD individuals (n = 5 for each group) was investigated. IFN-DCs from active TB patients (TB-DCs), compared to LTBI (LTBI-DCs) and HD (HD-DCs) counterparts, exhibited significant lower levels of both BDCA-3 and CD123 whereas the down-modulation of the BDCA-1 myeloid marker was comparable in both *Mtb* infected groups compared to HD (Fig 1C). Moreover, these cells exhibited considerably decreased CD80 levels mainly compared to HD-DCs, whereas CD86 levels were similar in all three groups (S2 Fig). All these data suggest a DC differentiation impairment mainly in charge of active TB patients. The molecular bases of altered differentiation of TB-DCs were investigated by using gene expression profile analysis (GEP). 288 genes were found differentially expressed in IFN-DCs derived from TB, HD and LTBI subjects, (*P*<0.005) (S1 Table). In particular, 146 and 102 genes were exclusively modulated in TB-DCs and LTBI-DCs, respectively, *versus* HD-DCs, whereas 14 genes fully distinguished the diverse expression program of these two populations (*P*<0.005) (Fig 2A, S3 Fig). The Principal Component Analysis (PCA) showed that both TB-DCs and HD-DCs clustered in two distinct groups well separated by PC1, whereas LTBI-DCs mostly exhibited an intermediate distribution (Fig 2B). Consistently, unsupervised tree clustering analysis highlighted that, while TB-DCs clustered significantly distant from HD-DCs, LTBI-DCs segregated intermediately between HD-DCs and TB-DCs, although closer to the latter (Fig 2C). These findings indicate that while a limited alteration of gene expression occurs also during IFN-α-driven differentiation of DCs from LTBI individuals, a profound gene expression modification associates with TB. The scatter plot comparison of transcriptional profiles showed a specific set of genes significantly down-modulated in active TB-DCs compared to HD-DCs (Fig 3A). Likewise, the same set of genes assigned LTBI-DCs in an intermediate position compared to the other two populations, accounting for their milder divergence from HD-DCs (Fig 3B). Accordingly, although IFN-DCs from both infected cohorts shared a down-modulation of the same functional genes, the degree of such alteration was significantly different in TB-DCs, displaying highly compromised expression of selected genes, also implicated in DC activation and Ag presentation such as *IRF4*, *CD80*, *CD1a* and *CD1c* (S2 Table, S4 Fig). At the same extent, a significant down-modulation of IFN-linked genes [31], such as *OASL*, *STAT-1*, *CXCL10*, *IL-15*, *TRIM22*, was observed specifically in TB-DCs compared to HD-DCs. Conversely, LTBI-DCs retained clear-cut expression of some IFN-inducible genes such as *TRIM22* or *IL-15* (Fig 3C, S3 Table). Of interest, the expression of transcription factors regulating the development of DC precursors as well as cDC and pDC subsets was also found to be significantly altered in TB-DCs (Fig 3D). Next, pairwise comparisons using GSEA across the three groups of IFN-DCs identified 4 GeneSets significantly enriched in HD-DCs compared to TB-DCs. Beyond the expected deficiency for IFN-α/β gene signature, TB-DCs were impaired for gene sets linked to DC maturation and function needed to be activated upon *Mtb* infection, such as LPS-induced DC maturation, *Mtb*-exposed DC and Ag processing/cross-presentation gene signatures (Fig 4A). Of interest, LTBI-DCs, compared to HD-DCs, displayed lower decrement of all gene sets, resulting more expressed compared to TB-DC counterpart (Fig 4B). To confirm IFN-α-related functional pathways and gene networks impairment in DCs from active TB patients, genes modulated in TB-DCs compared to HD counterpart were further mapped by IPA. Two main GO-functional terms were annotated in TB-DCs as functionally impaired: “activation dendritic cells” and “apoptosis dendritic cells” (Fig 5A); moreover, other canonical pathways, including Ag presentation and apoptosis of APC, were found affected in these cells (Fig 5B, S4 Table). IPA analysis also allowed to identify the main gene network dysregulated in TB-DCs and to denote main regulators, such as *IRF-1*, *IRF-5*, *IRF-7* and *TLR3*, whose altered expression may be associated to impaired functional pathways (Fig 5C). Altogether, IPA analysis confirmed defective functionality of selected DC signaling pathways in IFN-DCs from active TB patients and disclosed the potential alteration of key genes, governing additional signals such as apoptosis [32].

### IFN-DCs are poor stimulators of *Mtb*-specific T-cells in active TB patients

**Fig 6:**
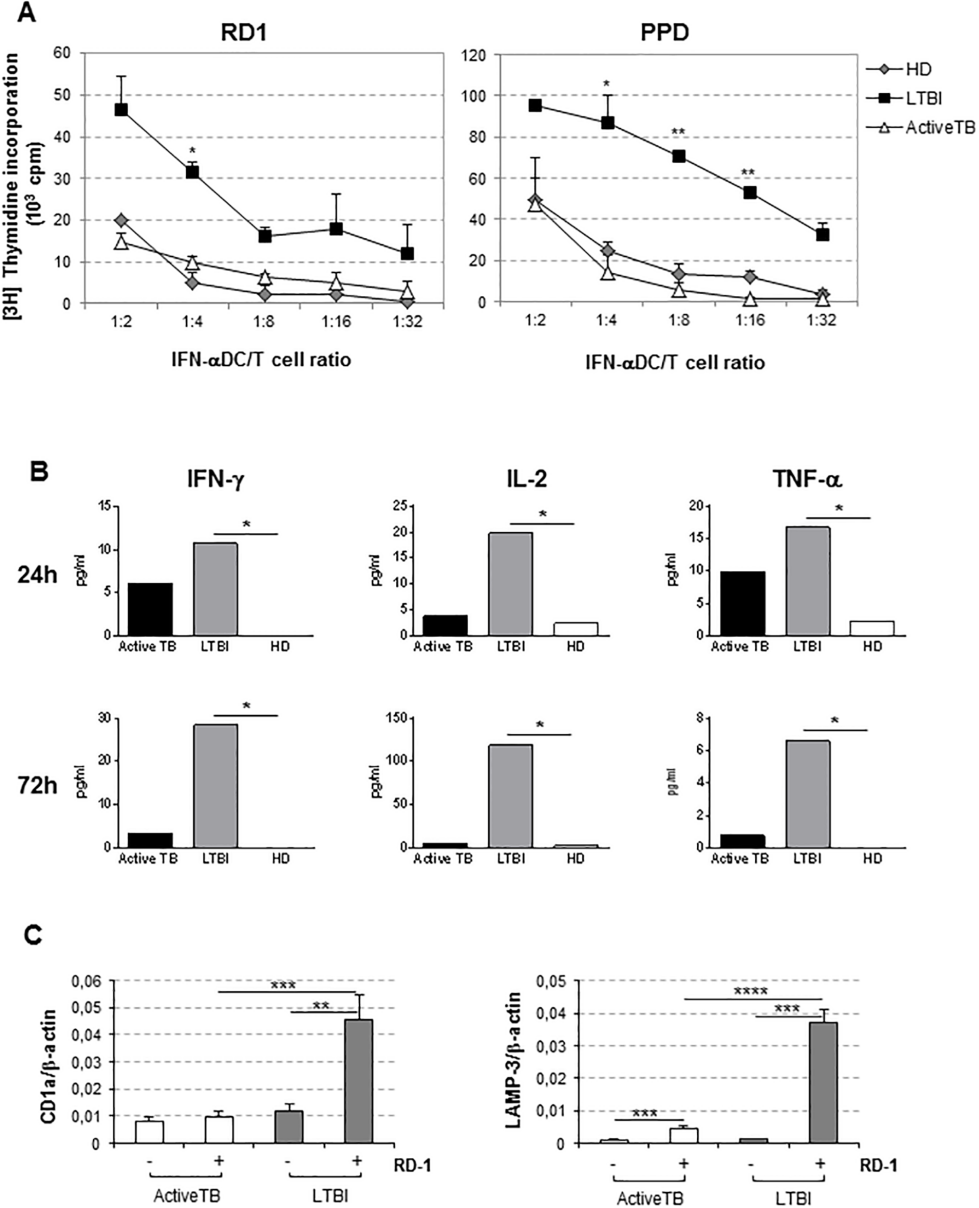
Defective T cell response to *Mtb* Ag stimulation in active TB patients. *A*, IFN-DCs generated from active TB, LTBI and HD individuals were pulsed for 24 hr with RD1 or PPD *Mtb* Ags and co-cultured with autologous T cells at indicated DC:T cell ratio. Data represent the mean cpm ± SEM of three independent experiments (N = 3; n = 3). *P* values refer to TB-DC and LTBI-DC comparison (Mann-Whitney test). *B*, Analysis of cytokine produced by T cells after stimulation for 24 or 72 hr with autologous RD1-loaded IFN-DCs. Cell culture supernatants were tested by flow cytometry using a cytokine bead array kit. *C*, CD1a and LAMP3 transcripts were quantified by qRT-PCR in IFN-DCs from active TB and LTBI subjects upon 24 hr RD1 stimulation and normalized to β-actin. Means ± SEM are shown (Mann-Whitney test). *P* values: *<0.05, **<0.01, ***<0.001.

To investigate whether impaired TB-DCs could affect *Mtb*-specific T cell activation capacity, we performed autologous stimulation of T cells with RD1 or PPD-loaded IFN-DCs. Of interest, we found that, after stimulating with both Ags, while LTBI-DCs induced higher levels of T cell proliferation compared to HD-DCs and TB-DCs in a dose-dependent manner, TB-DCs exhibited the complete loss of this ability and were unable to stimulate T cell proliferation as much as HD-DCs (Fig 6A). SEB-induced responses indicated residual functional capability of TB-DCs and the high propensity to sense stimuli of LTBI-DCs along with the functional competence of T cells from both active TB and LTBI individuals (S5 Fig). Accordingly, high levels of IFN-γ, IL-2 and TNF-α were produced over time by T cells from LTBI individuals when stimulated with RD1-loaded IFN-DCs, whereas active TB-derived T cells, after early moderate cytokine production, rapidly lost this capability (Fig 6B). Of interest, LTBI-DCs, compared to TB-DCs, were also found to up-modulate CD1a and LAMP-3 expression upon exposure to RD1 Ag (Fig 6C).

## Discussion

To date, the role of human DC subsets in instructing immunity of TB patients against *Mtb* compared to LTBI individuals remains largely unknown. Here, we report two novel findings: i) the impairment of selected DC subsets in individuals with active TB compared to LTBI, that conversely result broadly activated and prompted to stimulate T cell response; ii) the role of IFN-α in dictating DC developmental competence to stimulate *Mtb* Ag-specific immune response.

A high rate of mDCs, that may cooperate with pDCs in inducing CD4^+^ T cells [8], has been described as an important biomarker associated with LTBI [6]. Here, we reported a specific deficiency of peripheral BDCA3^+^ mDC2 and CD123^+^ pDCs populations in TB patients that, along with unchanged CD11c^+^ mDC1, show the same levels of CD80 and CD86 maturation markers as the HD counterpart. On the contrary, LTBI individuals exhibit broadly the same proportions of CD11c^+^ mDC1, BDCA3^+^ mDC2 and CD123^+^ pDCs as much as HD subjects, but significantly increased levels of CD80 or CD86. These data underline the impairment of DC compartment in TB patients compared to the LTBI counterpart, which results characterized by an activated cell phenotype. The specific defects of DC subsets deputed to Ag presentation in active TB patients underlies the incapability of these patients to develop an efficacious immunity according to the recent finding on the key role of pulmonary CD103^+^ DCs for developing host adaptive immune response after exposure to *Mtb* [33].

Within the DC network, monocyte-derived DCs are considered an extremely plastic DC subtype induced upon inflammation [9]. In particular, IFN-DCs, generated from monocytes after IFN-α exposure, share developmental and functional programs with pDCs [13] as well as some markers, such as BDCA3, with mDC2 (unpublished data) and highly resemble naturally occurring DCs, induced *in vivo* by infections and other danger stimuli [12]. We demonstrated that the peculiar functionality of IFN-DCs depends from the strong capability of IFN-I to induce developmental maturation of immature committed DCs driving their commitment in cross-presenting Ags and stimulating T cells [34]. Among these features, BDCA3 expression in human DCs associates with potent Ag presenting capability [35]. Noteworthy, IFN-DCs derived from active TB patients exhibit an altered developmental impairment outlined by very low levels of BDCA1, BDCA3, CD123 and CD80 molecules. Differently the IFN-DCs from LTBI individuals display BDCA3 expression similar to HD and higher levels of CD123 compared to HD. Altogether, these data suggest that the differential expression of BDCA1, BDCA3 and CD123 may associate to TB stages identification.

Activated neutrophil-driven IFN-I and type II IFN (IFN-II) signatures correlate with TB severity [17]. Importantly, IFN-I and IFN-II share common signals and often cooperate in inducing effective immunity. While the protective function of IFN-II against *Mtb* is well known, IFN-I role remains controversial [15]. IFN-I are pivotal for the activation of adaptive immune cell responses through the modulation of DC activity [34]. Therefore, IFN-I control of DC compartment acts at different levels: i) promotion of differentiation of precursor cells into DCs [13]; ii) activation of immature committed DCs licensing their capability to stimulate T cells [36]; iii) endorsement of DC behavior to cross-present Ags to CD8^+^ T cells [37]. For the developmental point of view IFN-DCs resemble natural occurring Type-1 polarized DCs, whose role as a potent immunogen against *Mtb* has been recently highlighted [38]. Noteworthy, TB-DCs exhibit striking down-modulation of key factors governing DC precursors development as well as cDC and pDC subsets, confirming their incapability to acquire some DC functions. In addition, due MHC-I and -II rearrangement, IFN-DCs possess high capability to phagocyte Ags thus inducing both CD8^+^ T cell cross-priming and CD4^+^ T cell activation [10, 26, 39].

At molecular level, IFN-DCs derived from TB patients exhibit high modulation of gene expression compared to that found in HD as well as in LTBI subjects. In particular, LTBI-DCs and TB-DCs share some degree of alterations, but these latter profoundly diverge for many signals. Among the commonly modulated genes there are *CCL22*, driver of Th22 cell recruitment and differentiation in response to TB Ags, *CCL17*, involved in chemokine-mediated response to *Mtb* infection, and *TLR3*, implicated in autophagy-mediated *Mtb* elimination in macrophages [40, 41]. Of note, a distinctive trait of LTBI-DCs is the retained capability to express some IFN-linked genes, such as *IL-15*, regulator of *Mtb*-specific CD8^+^ T effectors [42], that conversely results impaired in TB-DCs. Among the genes markedly compromised in TB-DCs, some are implicated in Ag presentation, such as *IRF-4*, which controls the MHC-II-master regulator CIITA [43], and *CD80*, a costimulatory molecule pivotal for lung immune cell recruitment and bacterial control [44]. The two major members of MHC-I-like CD1 family, *CD1a* and *CD1c*, implicated in processing and presentation of mycobacterial lipid Ags able to elicit cytotoxic and IFN-γ/TNF-β-producing CD1-restricted T cells [45], were also found defective in TB-DCs. Interestingly, upon exposure to *Mtb* Ags, only LTBI-DCs up-regulated *CD1a* expression. The further molecular dissection by GSEA revealed that TB-DCs crucially own impaired gene sets associated to DC capability to undergo phenotypic and functional maturation upon *Mtb* exposure as well as Ag processing and cross-presentation. These features along with altered “apoptosis” and “activation of dendritic cells” pathways disclosed by IPA studies, account for impaired IFN-driven functional signals in TB-DCs.

The outcome of DC interaction with *Mtb* remains unclear [46]. Many reports underlined DC impairment upon bacteria encountering, whereas others reported the unique properties of DCs in presenting *Mtb* Ags to T cells in both MHC-I- and MHC-II-dependent manner [3]. Certainly, a proper T cell response is necessary to determine whether *Mtb* infection resolves, progresses to LTBI or develops into active TB [47]. The frequency and phenotype of T cells have been suggested to distinguish between active disease and latent infection as activated *Mtb*-specific CD4^+^ T cells are higher in active TB than LTBI [48, 49]. Specific phenotypic and functional signatures of CD8^+^ T cells have also been found different between TB and LTBI individuals [50–53]. Here, we propose that the functional incompetence of TB-DCs is based on IFN-driven altered cellular signals. This assumption is further confirmed by the fully incapability of RD1 or complex PPD-loaded TB-DCs to induce proliferation of autologous Ag-specific T cells while retaining capability to respond to SEB. Conversely, LTBI-DCs are proficient to induce Ag-specific T cells proliferation and IFN-γ, IL2, and TNF-α production, key cytokines produced by both CD4^+^ and CD8^+^ T cells during immune response to *Mtb* [54]. In this context, we cannot exclude as well that a modulation of the PD-1/PD-L1 axis between LTBI-DCs and TB-DCs may play a role [55].

Taken together, our results describe an important role of IFN-I in the induction of immunity against *Mtb* infection. Although several reports underlined that high levels of IFN-I play a harmful role in *Mtb* infection [16], it is crucial to keep in mind that IFN-I signaling is essential for the induction of some signals, such as IL-12-mediated IFN-γ priming in the bone marrow or the generation of tertiary lymphoid structures, well-known to play an antibacterial role [56–58]. Here, we convey evidence supporting that the preservation of the IFN-I signature in DCs is essential for eliciting *Mtb*-specific T cell response as it occurs in LTBI subjects. We envisage that in the slighting game played by IFN-I during TB the cellular source as well as the magnitude and timing of IFN-I production may play a role [59]. A specific role of IFN-α subtype is also foreseen for both Ag-specific T cell stimulation and cytokine production implicated in the immune response against infection [59]. Hence, the dichotomy of IFN-I role in *Mtb* infection needs future investigation.

## Supporting information

S1 Fig Gating strategy. (TIF)

S2 Fig Analysis of DC differentiation markers. (TIF)

S3 Fig Clustering of differentially expressed genes. (TIF)

S4 Fig Microarray data validation. (TIF)

S5 Fig T lymphocytes from active TB patients retain their capability to response to Staphylococcal enterotoxin B (SEB). (TIF)

S1 Table Differentially expressed genes by comparing IFN-DCs derived from active TB, LTBI and HD individuals. (PDF)

S2 Table Genes differentially expressed among TB-DCs, LTBI-DCs and HD-DCs. (PDF)

S3 Table Relative expression level of IFN genes. (PDF)

S4 Table Biological function categories modulated in TB-DCs. (PDF)
