# Supplementary figures and images for: Impaired IFN-α-mediated signal in dendritic cells differentiates active from latent tuberculosis
Source: PLoS One. 2018 Jan 10;13(1):e0189477. doi: 10.1371/journal.pone.0189477 (PMC5761858; doi:10.1371/journal.pone.0189477)

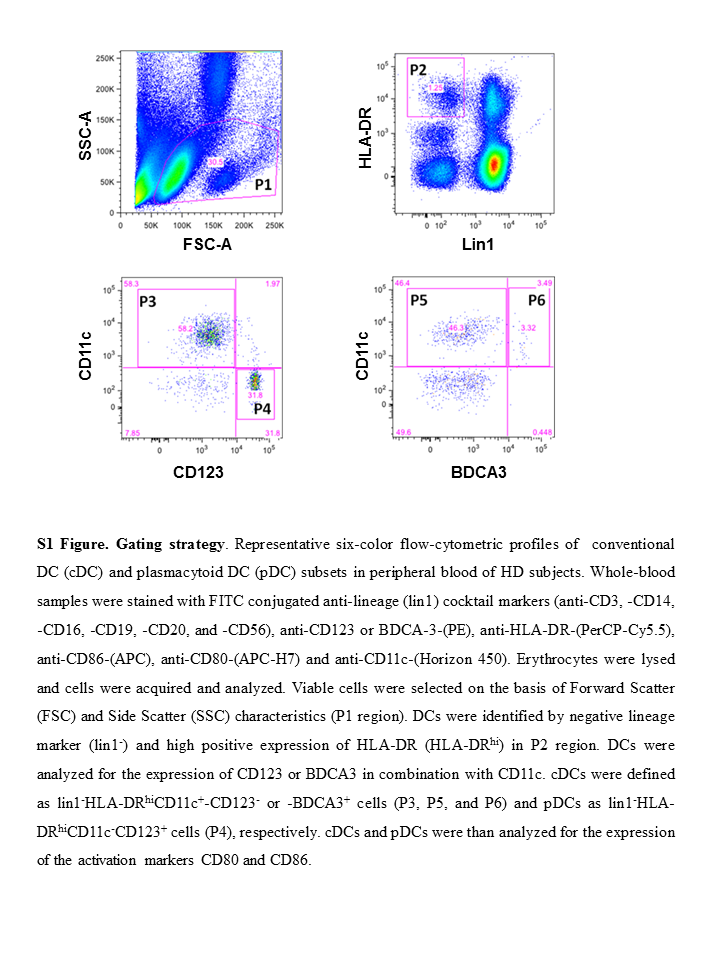

Supplement: S1 Fig — (TIF) [file pone.0189477.s001.tif]

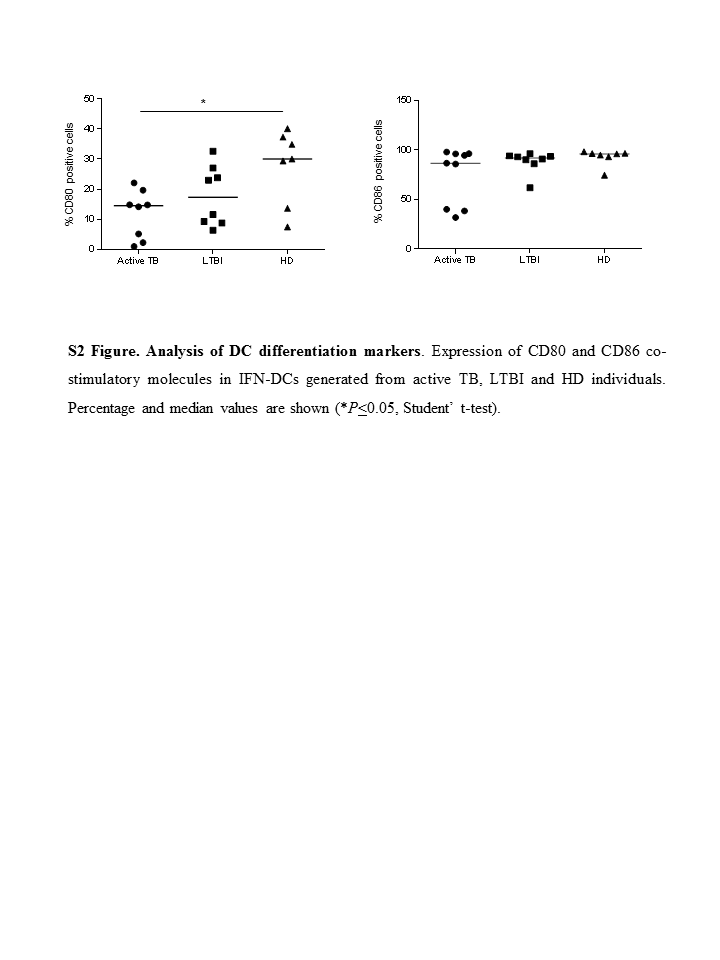

Supplement: S2 Fig — (TIF) [file pone.0189477.s002.tif]

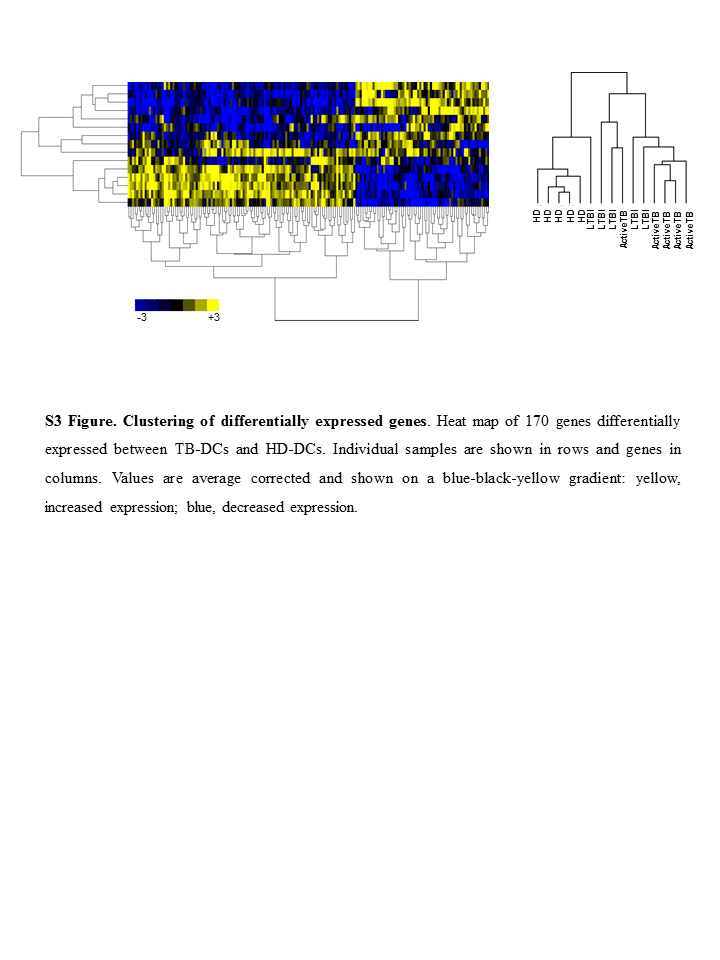

Supplement: S3 Fig — (TIF) [file pone.0189477.s003.tif]

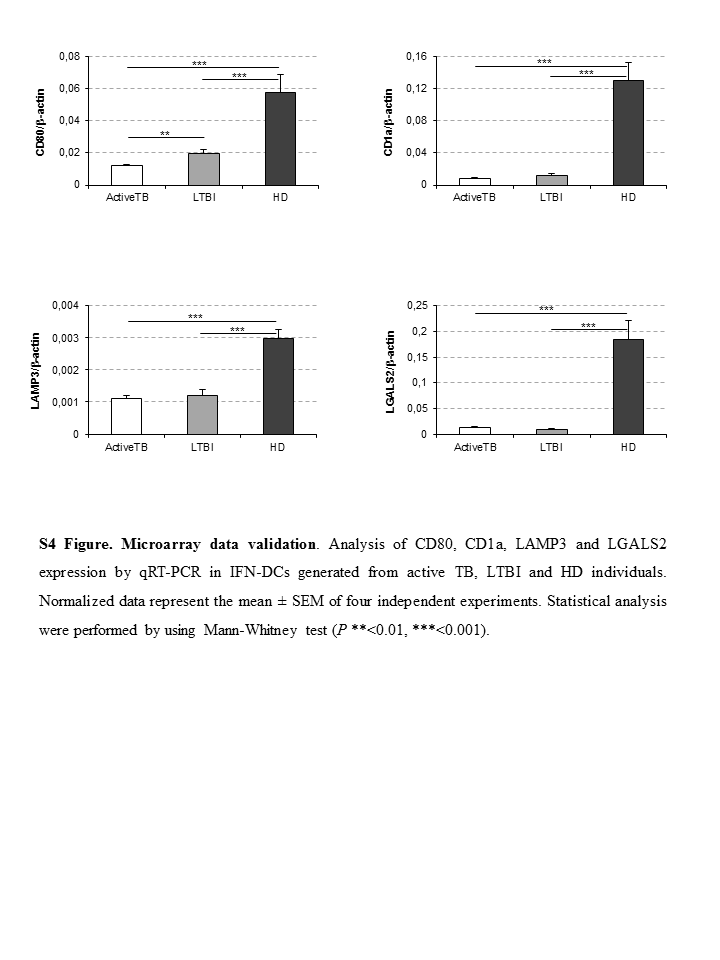

Supplement: S4 Fig — (TIF) [file pone.0189477.s004.tif]

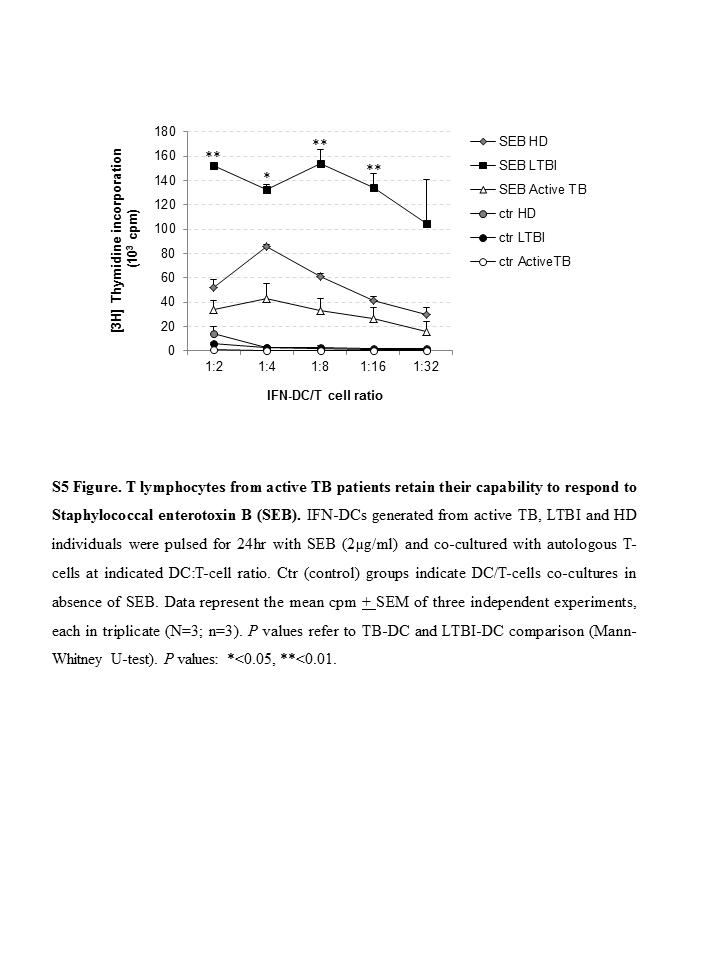

Supplement: S5 Fig — (TIF) [file pone.0189477.s005.tif]
