# Supplementary material for: Impaired IFN-α-mediated signal in dendritic cells differentiates active from latent tuberculosis
Source: PLoS One. 2018 Jan 10;13(1):e0189477. doi: 10.1371/journal.pone.0189477 (PMC5761858; doi:10.1371/journal.pone.0189477)
Supplement: S1 Table — (PDF) [file pone.0189477.s006.pdf]

S1 Table. Differentially expressed genes among IFN-DCs derived from ActiveTB, LTBI and HD individuals.

Gene symbol	Description	Active TB *vs* LTBI	Active TB *vs* HD	LTBI *vs* HD
**ACBD5**	-		X	
**ADAMTS9**	a disintegrin-like and metalloprotease (reprolysin type) with thrombospondin type 1 motif, 9			X
**ADCK5**	aarF domain containing kinase 5		X	
**ADPN**	adiponutrin			X
**ALDH1A2**	aldehyde dehydrogenase 1 family, member A2	X		
**ANPEP**	alanyl (membrane) aminopeptidase (aminopeptidase N, aminopeptidase M, microsomal aminopeptidase, CD13, p150)		X	X
**AP2S1**	adaptor-related protein complex 2, sigma 1 subunit		X	
**APBB2**	amyloid beta (A4) precursor protein-binding, family B, member 2 (Fe65-like)	X		
**APR2_HUMAN**	apoptosis related protein			X
**ARHGAP29**	PTPL1-associated RhoGAP 1			X
**ARHGAP31**	KIAA1204 protein		X	
**ARHGEF6**	Rac/Cdc42 guanine nucleotide exchange factor (GEF) 6		X	
**ATBF1**	AT-binding transcription factor 1			X
**AZGP1**	alpha-2-glycoprotein 1, zinc	X		
**BCAR1**	breast cancer anti-estrogen resistance 1			X
**BDKRB2**	bradykinin receptor B2		X	
**BID**	BH3 interacting domain death agonist		X	
**BTN1A1**	butyrophilin, subfamily 1, member A1		X	
**C13orf15**	response gene to complement 32		X	
**C14orf160**	chromosome 14 open reading frame 160			X
**C17orf62**	hypothetical protein MGC4368		X	
**C1orf113**	hypothetical protein FLJ22938		X	
**C20orf127**	chromosome 20 open reading frame 127			X
**C22orf36**	similar to hypothetical protein LOC192734			X
**C6orf223**	-			X
**C6orf62**	chromosome 6 open reading frame 62		X	
**C9orf58**	chromosome 9 open reading frame 58			X
**CARD16**	CARD only protein		X	
**CCL17**	chemokine (C-C motif) ligand 17		X	
**CCL22**	chemokine (C-C motif) ligand 22		X	
**CCNL1**	cyclin L1		X	
**CD1A**	CD1A antigen, a polypeptide		X	
**CD1C**	CD1C antigen, c polypeptide		X	
**CD80**	CD80 antigen (CD28 antigen ligand 1, B7-1 antigen)		X	X
**CDC40**	cell division cycle 40 homolog (yeast)		X	
**CDC42EP1**	CDC42 effector protein (Rho GTPase binding) 1		X	
**CDC7**	CDC7 cell division cycle 7 (S. cerevisiae)			X
**CDCA7**	cell division cycle associated 7		X	
**CEACAM1**	carcinoembryonic antigen-related cell adhesion molecule 1 (biliary glycoprotein)			X

Gene symbol	Description	Active TB *vs* LTBI	Active TB *vs* HD	LTBI *vs* HD
**CHCHD5**	coiled-coil-helix-coiled-coil-helix domain containing 5			X
**CHEK1**	CHK1 checkpoint homolog (S. pombe)		X	X
**CISH**	cytokine inducible SH2-containing protein		X	
**CLECSF8**	C-type (calcium dependent, carbohydrate-recognition domain) lectin, superfamily member 8		X	
**CMAS**	cytidine monophosphate N-acetylneuraminic acid synthetase		X	X
**COBL**	cordon-bleu homolog (mouse)			X
**COL5A2**	collagen, type V, alpha 2		X	
**CPB2**	carboxypeptidase B2 (plasma, carboxypeptidase U)			X
**CRHR1**	corticotropin releasing hormone receptor 1		X	X
**CST1**	cystatin SN		X	
**CST3**	cystatin C (amyloid angiopathy and cerebral hemorrhage)		X	
**CST6**	cystatin E/M			X
**CTRL**	proteasome (prosome, macropain) subunit, beta type, 10			X
**CTSL2**	cathepsin L2		X	
**CUL5**	cullin 5			X
**DAF**	decay accelerating factor for complement (CD55, Cromer blood group system)			X
**DCOP_HUMAN**	-		X	
**DCXR**	dicarbonyl/L-xylulose reductase		X	
**DDX58**	DEAD (Asp-Glu-Ala-Asp) box polypeptide 58		X	
**DEPDC1**	DEP domain containing 1			X
**DEPDC1B**	DEP domain containing 1B		X	
**DKK1**	dickkopf homolog 1 (Xenopus laevis)		X	X
**DNAJC6**	DnaJ (Hsp40) homolog, subfamily C, member 6		X	
**DNASE1L3**	deoxyribonuclease I-like 3	X		
**DNMT3B**	DNA (cytosine-5-)-methyltransferase 3 beta			X
**DRAP1**	DR1-associated protein 1 (negative cofactor 2 alpha)		X	
**DRD1**	dopamine receptor D1		X	
**E2F2**	E2F transcription factor 2			X
**EDF1**	endothelial differentiation-related factor 1		X	
**EIF4EBP1**	eukaryotic translation initiation factor 4E binding protein 1		X	
**EPAS1**	endothelial PAS domain protein 1		X	
**EVA1**	epithelial V-like antigen 1			X
**FAM29A**	family with sequence similarity 29, member A		X	
**FAM73A**	FLJ35093 protein		X	
**FARS1**	phenylalanine-tRNA synthetase 2 (mitochondrial)			X
**FEZF1**	similar to zinc finger protein 312; forebrain embryonic zinc finger			X
**FGB**	fibrinogen, B beta polypeptide			X
**FGF13**	fibroblast growth factor 13			X
**FGL1**	fibrinogen-like 1			X
**FJX1**	four jointed box 1 (Drosophila)			X
**FMNL1**	formin-like 1		X	

Gene symbol	Description	Active TB *vs* LTBI	Active TB *vs* HD	LTBI *vs* HD
**FPRL2**	formyl peptide receptor-like 2		X	X
**GAB4**	-		X	
**GALNAC4S-6ST**	B cell RAG associated protein		X	X
**GAS2**	growth arrest-specific 2			X
**GCM2**	glial cells missing homolog 2 (Drosophila)			X
**GDAP2**	ganglioside induced differentiation associated protein 2		X	
**GLT8D1**	glycosyltransferase 8 domain containing 1			X
**GPR157**	-		X	
**GPX7**	glutathione peroxidase 7		X	
**GRB10**	growth factor receptor-bound protein 10		X	
**GRIK2**	glutamate receptor, ionotropic, kainate 2	X		
**GTF3C1**	general transcription factor IIIC, polypeptide 1, alpha 220kDa		X	
**HAS3**	hyaluronan synthase 3			X
**HDAC1**	histone deacetylase 1			X
**HDAC3**	histone deacetylase 3		X	
**HECA**	headcase homolog (Drosophila)	X		
**HERC6**	hect domain and RLD 6		X	
**HEXDC**	hypothetical protein FLJ23825		X	
**HIF1AN**	hypoxia-inducible factor 1, alpha subunit inhibitor		X	
**HIG2_HUMAN**	-		X	
**HLA-DQA2**	major histocompatibility complex, class II, DQ alpha 2		X	
**HLA-DRB2**	major histocompatibility complex, class II, DR beta 1			X
**HMMR**	hyaluronan-mediated motility receptor (RHAMM)		X	
**HPS1**	-		X	
**HPX**	hemopexin			X
**HTR4**	5-hydroxytryptamine (serotonin) receptor 4		X	X
**ICMT**	-		X	
**IFI35**	interferon-induced protein 35		X	
**IFIT3**	interferon-induced protein with tetratricopeptide repeats 3		X	
**IL17RC**	interleukin 17 receptor C		X	
**IQCF1**	IQ motif containing F1			X
**IRF4**	interferon regulatory factor 4	X	X	
**ITM2A**	integral membrane protein 2A			X
**KAL1**	Kallmann syndrome 1 sequence			X
**KAZALD1**	hypothetical gene supported by BC012394; BC053611		X	
**KIAA1787**	-		X	
**KLHL12**	kelch-like 12 (Drosophila)		X	
**KNG1**	kininogen 1			X
**LAMA1**	laminin, alpha 1			X
**LAMB1**	laminin, beta 1		X	
**LAMP3**	lysosomal-associated membrane protein 3		X	

Gene symbol	Description	Active TB *vs* LTBI	Active TB *vs* HD	LTBI *vs* HD
**LGALS1**	lectin, galactoside-binding, soluble, 1 (galectin 1)		X	
**LGALS2**	lectin, galactoside-binding, soluble, 2 (galectin 2)		X	
**LILRB1**	leukocyte immunoglobulin-like receptor, subfamily B (with TM and ITIM domains), member 1			X
**LILRB3**	leukocyte immunoglobulin-like receptor, subfamily B (with TM and ITIM domains), member 3			X
**LLGL2**	lethal giant larvae homolog 2 (Drosophila)			X
**LOC349683**	-		X	
**LRIG3**	leucine-rich repeats and immunoglobulin-like domains 3		X	
**LRP8**	-			X
**LSM4**	LSM4 homolog, U6 small nuclear RNA associated (S. cerevisiae)		X	
**M6PR**	mannose-6-phosphate receptor (cation dependent)		X	
**MAD2L2**	MAD2 mitotic arrest deficient-like 2 (yeast)		X	
**MAGEC1**	melanoma antigen family C, 1		X	
**MAPK14**	mitogen-activated protein kinase 14		X	
**MARK2**	-	X		
**MBD3L1**	methyl-CpG binding domain protein 3-like 1		X	
**MCM5**	MCM5 minichromosome maintenance deficient 5, cell division cycle 46 (S. cerevisiae)		X	
**MCOLN1**	mucolipin 1		X	
**MET**	met proto-oncogene (hepatocyte growth factor receptor)		X	X
**MICB**	MHC class I polypeptide-related sequence B	X		
**MOV10**	-	X		
**MOXD1**	monooxygenase, DBH-like 1			X
**MPEG1**	-			X
**MT1P2**	-		X	X
**MT2A**	hypothetical gene supported by X97260; BC070289		X	X
**MTA3**	metastasis associated 1 family, member 3			X
**MTMR11**	cisplatin resistance associated			X
**MTUS1**	mitochondrial tumor suppressor 1		X	
**NAPE-PLD**	N-acyl-phosphatidylethanolamine-hydrolyzing phospholipase D		X	
**NCF2**	neutrophil cytosolic factor 2 (65kDa, chronic granulomatous disease, autosomal 2)		X	
**NCF4**	neutrophil cytosolic factor 4, 40kDa		X	
**NCR2**	-		X	
**NKD2**	-		X	
**NME3**	non-metastatic cells 3, protein expressed in		X	
**NME7**	non-metastatic cells 7, protein expressed in (nucleoside-diphosphate kinase)			X
**NMT2**	N-myristoyltransferase 2			X
**NP_001004322**	FLJ38717 protein		X	
**NP_076416**	HCV NS3-transactivated protein 2		X	X
**NP_077817**	p10-binding protein			X
**NP_079019**	hypothetical protein FLJ21934			X
**NP_115700**	hypothetical protein MGC13186			X

Gene symbol	Description	Active TB *vs* LTBI	Active TB *vs* HD	LTBI *vs* HD
**NP_620311**	apobec-1 complementation factor		X	
**NP_659461**	hypothetical protein FLJ31547		X	
**NP_689595**	hypothetical protein FLJ37953		X	
**NP_694540**	ovarian zinc finger protein			X
**NP_848603**	chromosome 6 open reading frame 1			X
**NP_848622**	solute carrier family 9, isoform 11		X	
**NP_857596**	-		X	
**NP_997377**	similar to bA110H4.2 (similar to membrane protein)	X		
**NR4A2**	nuclear receptor subfamily 4, group A, member 2			X
**O9I1_HUMAN**	olfactory receptor, family 9, subfamily I, member 1			X
**OMA1**	-	X		X
**OR13C5**	olfactory receptor, family 13, subfamily C, member 5		X	X
**PA2G4**	-		X	
**PADI4**	peptidyl arginine deiminase, type IV			X
**PAM**	peptidylglycine alpha-amidating monooxygenase			X
**PANK1**	pantothenate kinase 1			X
**PCDHB8**	protocadherin beta 8		X	
**PFN1**	profilin 1		X	
**PGA5**	pepsinogen 5, group I (pepsinogen A)		X	
**PGBD5**	piggyBac transposable element derived 5			X
**PHF20L1**	PHD finger protein 20-like 1			X
**PILRB**	paired immunoglobin-like type 2 receptor beta		X	
**PKN3**	protein kinase N3			X
**PLAC8**	placenta-specific 8			X
**PODXL**	podocalyxin-like			X
**PP**	pyrophosphatase (inorganic)		X	
**PPM1L**	protein phosphatase 1 (formerly 2C)-like			X
**PRKCABP**	protein kinase C, alpha binding protein		X	
**PRKX**	-			X
**PRM2**	protamine 2	X		
**PSCD4**	pleckstrin homology, Sec7 and coiled-coil domains 4		X	
**PTGES**	prostaglandin E synthase		X	
**PTMS**	parathymosin		X	
**PTPNS1**	protein tyrosine phosphatase, non-receptor type substrate 1		X	
**PUS1**	pseudouridylate synthase 1		X	
**Q8N985**	-			X
**Q8NAC9**	KIAA0540 protein			X
**Q8NAN8**	-		X	
**Q8NB05**	-	X		
**Q8WY58**	hypothetical protein LOC201175		X	
**Q96HN1**	-	X	X	

Gene symbol	Description	Active TB *vs* LTBI	Active TB *vs* HD	LTBI *vs* HD
**Q9P1F5**	-			X
**RBMY2EP**	-			X
**RFC5**	replication factor C (activator 1) 5, 36.5kDa		X	
**RGNEF**	-		X	X
**RGS14**	regulator of G-protein signalling 14		X	
**RGS19**	regulator of G-protein signalling 19		X	
**RHOJ**	-		X	
**RNF128**	ring finger protein 128		X	
**RPL12**	ribosomal protein L12		X	X
**RPL23AP82**	similar to RPL23AP7 protein			X
**RPS2P8**	similar to ribosomal protein S2; 40S ribosomal protein S2		X	
**RRAS2**	related RAS viral (r-ras) oncogene homolog 2			X
**RXRG**	retinoid X receptor, gamma		X	
**S100A6**	S100 calcium binding protein A6 (calcyclin)		X	
**SAH**	SA hypertension-associated homolog (rat)		X	
**SCCPDH**	CGI-49 protein		X	X
**SDF2L1**	stromal cell-derived factor 2-like 1		X	
**SELRC1**	hypothetical protein FLJ12439		X	
**SEMA3C**	sema domain, immunoglobulin domain (Ig), short basic domain, secreted, (semaphorin) 3C			X
**SERPINC1**	serine (or cysteine) proteinase inhibitor, clade C (antithrombin), member 1		X	
**SFTPB**	surfactant, pulmonary-associated protein B		X	
**SGCE**	sarcoglycan, epsilon		X	X
**SGOL1**	-		X	
**SH3BGRL2**	SH3 domain binding glutamic acid-rich protein like 2			X
**SH3GL2**	-		X	
**SIAE**	cytosolic sialic acid 9-O-acetylesterase homolog			X
**SLC13A4**	-		X	
**SLC16A9**	solute carrier family 16 (monocarboxylic acid transporters), member 9		X	
**SLC35F2**	solute carrier family 35, member F2			X
**SLC43A2**	solute carrier family 43, member 2		X	
**SLC7A11**	-			X
**SMC1L1**	SMC1 structural maintenance of chromosomes 1-like 1 (yeast)		X	
**SNX25**	sorting nexin 25			X
**SON**	SON DNA binding protein		X	
**SPANXD**	SPANX family, member D			X
**SPATA4**	spermatogenesis associated 4			X
**SPC25**	kinetochore protein Spc25			X
**SPG11**	hypothetical protein FLJ21439		X	
**SPHK1**	sphingosine kinase 1			X
**STAB1**	stabilin 1			X
**STAT4**	signal transducer and activator of transcription 4		X	

Gene symbol	Description	Active TB *vs* LTBI	Active TB *vs* HD	LTBI *vs* HD
**STK10**	serine/threonine kinase 10			X
**SVIL**	supervillin	X	X	
**SYBL1**	synaptobrevin-like 1		X	
**TACSTD1**	tumor-associated calcium signal transducer 1		X	X
**TALDO1**	transaldolase 1			X
**TAOK3**	TAO kinase 3		X	
**TBCC**	tubulin-specific chaperone c		X	
**TFEB**	transcription factor EB			X
**THY1**	Thy-1 cell surface antigen			X
**TLR3**	toll-like receptor 3		X	X
**TM4SF11**	transmembrane 4 superfamily member 11 (plasmolipin)		X	
**TM7SF1**	transmembrane 7 superfamily member 1 (upregulated in kidney)		X	
**TM7SF3**	transmembrane 7 superfamily member 3			X
**TMEM98**	DKFZP564K1964 protein		X	X
**TMTC1**	-		X	
**TNFRSF11A**	tumor necrosis factor receptor superfamily, member 11a, activator of NFKB		X	
**TNFSF10**	tumor necrosis factor (ligand) superfamily, member 10		X	
**TPI1**	triosephosphate isomerase 1		X	
**TPM1**	tropomyosin 1 (alpha)		X	
**TPST2**	tyrosylprotein sulfotransferase 2		X	
**TRADD**	TNFRSF1A-associated via death domain		X	
**TREM2**	triggering receptor expressed on myeloid cells 2		X	
**TRIM16**	similar to tripartite motif-containing 16; estrogen-responsive B box protein		X	
**TSC22D1**	transforming growth factor beta 1 induced transcript 4		X	
**TTC21B**	tetratricopeptide repeat domain 21B	X		X
**TYK2**	tyrosine kinase 2		X	
**TYR**	tyrosinase (oculocutaneous albinism IA)			X
**UNC93B1**	unc-93 homolog B1 (C. elegans)		X	
**URP2_HUMAN**	UNC-112 related protein 2		X	
**VPS28**	vacuolar protein sorting 28 (yeast)	X		
**WAC**	-			X
**WDR48**	WD repeat domain 48			X
**XIST**	-			X
**Y220_HUMAN**	-		X	
**YARS2**	CGI-04 protein			X
**ZFAND1**	hypothetical protein FLJ14007			X
**ZNF142**	zinc finger protein 142 (clone pHZ-49)		X	
**ZNF18**	zinc finger protein 18 (KOX 11)			X
**ZNF223**	zinc finger protein 223		X	
**ZNF416**	hypothetical protein FLJ20557			X
**ZNF521**	zinc finger protein 521			X

Gene symbol	Description	Active TB *vs* LTBI	Active TB *vs* HD	LTBI *vs* HD
**ZNF615**	zinc finger protein 615	X		
**ZNF682**	-		X	
**ZNRF1**	-			X
**ZWILCH**	Zwilch			X

Footnotes: X indicate the comparison groups in which the indicated genes are differentially expressed
