# Supplementary material for: Impaired IFN-α-mediated signal in dendritic cells differentiates active from latent tuberculosis
Source: PLoS One. 2018 Jan 10;13(1):e0189477. doi: 10.1371/journal.pone.0189477 (PMC5761858; doi:10.1371/journal.pone.0189477)
Supplement: S2 Table — (PDF) [file pone.0189477.s007.pdf]

S2 Table. Genes differentially expressed among TB-DCs, LTBI-DCs and HD-DCs

Genes	Active TB	LTBI	HD	*P**	*P°*	*P^*
**IFI35**	1.8	2.0	4.2	**0.0004249**	0.788434	0.0347008
**CCL22**	1.1	1.7	5.1	**0.0011122**	0.110047	0.0222189
**CCL17**	8.9	12.7	27.5	**0.0043973**	0.496959	0.0995179
**IFIT3**	3.9	4.4	9.7	**0.0009485**	0.726082	0.0210661
**CD80**	1.0	1.6	3.0	**8.92e-05**	0.053962	**0.0029461**
**TNFSF10**	7.5	12.8	23.7	**0.0034522**	0.123511	0.0299969
**IRF4**	0.9	2.1	2.7	**7.45e-05**	**0.0017076**	0.30211
**CD1a**	4.5	8.4	28.9	**0.000318**	0.21533	0.0301759
**CD1c**	9.4	17.7	43.0	**0.0044324**	0.288013	0.0875511
**TLR3**	2.1	2.0	4.4	**0.0024791**	0.944504	**0.0041855**
**HLA-DQA2**	21.4	28.8	50.5	**0.0004992**	0.944504	**0.0041855**
**LGALS2**	2.92	5.38	31.05	**0.000125**	0.391292	0.0082647
**LAMP3**	1.6	2.56	4.77	**0.0001184**	0.0088639	0.0120841

Relative expression levels and *P* values are indicated. Active TB, LTBI and HD indicate TB-DCs, LTBI-DCs and HD-DCs, respectively. * ActiveTB *vs* HD comparison. ° ActiveTB *vs* LTBI comparison. ^ LTBI *vs* HD comparison. In **bold**, statistically significant differences: $P \leq 0.005$.
