# Supplementary material for: Impaired IFN-α-mediated signal in dendritic cells differentiates active from latent tuberculosis
Source: PLoS One. 2018 Jan 10;13(1):e0189477. doi: 10.1371/journal.pone.0189477 (PMC5761858; doi:10.1371/journal.pone.0189477)
Supplement: S3 Table — (PDF) [file pone.0189477.s008.pdf]

S3 Table. Relative Expression level of IFN genes

Gene Symbol	Active TB	LTBI	HD
**IFI35**	0.85737	0.984356	2.07484
**IRF7**	2.861173	2.781195	3.545011
**OAS1**	3.297533	3.813172	4.146626
**BAZ1A**	0.230573	0.579528	0.773642
**STAT1**	2.931846	3.26565	4.12805
**GBP1**	-0.14399	-0.0061	0.090986
**IFIT3**	1.971148	2.124119	3.280094
**TDRD7**	0.972591	1.70536	1.870732
**TNFSF10**	2.911916	3.672478	4.568414
**C1orf38**	1.869304	2.10852	2.287971
**BST2**	-1.51567	-1.18735	-0.68666
**TRIM22**	2.781689	3.842806	3.666674
**OASL**	2.935888	3.53013	4.020926
**STX11**	1.601968	1.947235	2.834729
**LMO2**	1.212718	1.820563	1.835627
**SP110**	1.27387	0.857668	2.007371
**CASP1**	2.464106	3.423086	3.799689
**IFIT5**	1.466979	1.891759	2.515557
**RABGAP1L**	2.252348	3.041724	2.935615
**MX1**	3.864673	3.675852	4.517303
**IL15**	-0.43985	0.193476	0.4452
**TLR3**	1.050413	1.031107	2.148624
**PSMB8**	1.982478	1.889523	2.541505
**CXCL10**	2.385954	2.909307	4.03181
**ZBTB20**	-1.86492	-1.44267	-1.169
