# Supplementary material for: Impaired IFN-α-mediated signal in dendritic cells differentiates active from latent tuberculosis
Source: PLoS One. 2018 Jan 10;13(1):e0189477. doi: 10.1371/journal.pone.0189477 (PMC5761858; doi:10.1371/journal.pone.0189477)
Supplement: S4 Table — (PDF) [file pone.0189477.s009.pdf]

S4 Table. Biological function categories modulated in TB-DCs.

Function Annotation	P-Value	Activation z-score	# Molecules
**immune response of cells**	1,83E-05	-2,246	17
**activation of lymphocytes**	1,29E-05	-2,187	15
**response of antigen presenting cells**	2,68E-05	-1,830	9
**recruitment of T lymphocytes**	1,69E-03	-1,698	4
**engulfment of antigen presenting cells**	5,18E-04	-1,620	6
**immune response of antigen presenting cells**	5,93E-04	-1,561	7
**activation of dendritic cells**	1,98E-04	-1,524	6
**migration of antigen presenting cells**	4,04E-04	-1,501	7

Ingenuity pathway analysis (IPA) of genes selectively modulated in TB-DCs *versus* HD-DCs.
